# Nurse-led normalised advance care planning service in hospital and community health settings: a qualitative study

**DOI:** 10.1186/s12904-021-00835-x

**Published:** 2021-09-09

**Authors:** Se Ok Ohr, Peter Cleasby, Sarah Yeun-Sim Jeong, Tomiko Barrett

**Affiliations:** 1 HNE Nursing and Midwifery Research Centre, Conjoint Lecturer University of Newcastle, James Fletcher Campus, Gate Cottage, 72 Watt Street, Hunter New England Local Health District, Newcastle, NSW 2300 Australia; 2 Division of Aged, Subacute and Complex Care, PO Box 6088, Central Coast Local Health District, Long Jetty, NSW 2261 Australia; 3 School of Nursing and Midwifery, The University of Newcastle, 10 Chittaway Road, Ourimbah, NSW 2258 Australia; 4 Department of Aged Care Services, Wyong Hospital, PO Box 4200, Central Coast Local Health District, Lakehaven, NSW 2263 Australia

**Keywords:** Advance care planning, Advance care directives, Chronic diseases, Community, Hospital, Health service, Nurses

## Abstract

**Background:**

Advance Care Planning (ACP) by Registered Nurses (RNs) has been emerging. However, there is limited understanding about what RNs experience as they incorporate ACP into their practice. This study aimed to elicit the experiences of ACP RNs with the implementation of a normalised ACP (NACP) service in hospital and community care settings.

**Methods:**

A qualitative descriptive study invited four ACP RNs who delivered a nurse-led NACP for a 6 months duration at two hospital and two community health care settings in New South Wales (NSW), Australia. The experiences of the ACP RNs were captured through a semi-structured interview and weekly debriefing meetings. The interview recordings were transcribed verbatim and the minutes of weekly debriefing meetings were utilized. Data were analysed by two independent researchers using thematic analysis with the Normalisation Process Theory (NPT) as a methodological framework.

**Findings:**

The ACP RNs were females with a mean age of 43 years old. Their nursing experiences ranged 2 to 25 years but they had minimal experiences with ACP and had not attended any education about ACP previously. The following four themes were identified in the experiences of the ACP RNs; 1) Embracing NACP service; 2) Enablers and barriers related to patients and health professionals; 3) Enablers and barriers related to ACP RNs; and 4) What it means to be an ACP RN.

**Conclusion:**

The introduction of a NACP service into existing clinical systems is complex. The study demonstrated the capacity of RNs to engage in ACP processes, and their willingness to deliver an NACP service with a raft of locally specific enablers and barriers.

**Trial registration:**

The study was retrospectively registered with the Australian New Zealand Clinical Trials Registry (Trial ID: ACTRN12618001627246). The URL of the trial registry record

**Supplementary Information:**

The online version contains supplementary material available at 10.1186/s12904-021-00835-x.

## Background

Advance care planning is a process that involves people, their loved ones and health professionals to discuss about their values and consider the types of health care they would want to receive or decline if they become unable to make decisions for and by themselves [1] . Despite numerous attempts to promote ACP worldwide over the last two decades, the uptake of ACP remains low [2–4]. Consequently, increasing the uptake of ACP has been a focus of policy makers, health care professionals, and researchers [5–10]. In particular, whilst there has been consensus that nurses have crucial roles to play in ACP, there have been consistent reports that nurses perceived barriers despite their positive attitudes towards ACP [11–13]. This is of concern as it may imply a lack of commitment and action by nursing profession.

ACP is well known to be beneficial to individuals, their families, and health care professionals [11, 14–16]. However, while patients themselves can initiate ACP, there are many reasons why uptake is low [17]. Extensive literature has already identified that the reasons for the low uptake of ACP from patients’ and families’ perspective include inadequate awareness of ACP, societal reluctance to discuss end-of-life issues, time consuming, and complexity of form [17, 18]). The studies focused on the facilitators and barriers to ACP from health care professionals’ perspective report the lack of health professionals’ confidence and commitment [11, 19]. Despite the various challenges, one consensus is that health professionals are the ones to initiate ACP to assist patients [20]. Among health professionals, social workers have demonstrated a leadership in encouraging and facilitating ACP in Canada [21], and the United States of America (USA) [22]. General Practitioners in Australia also have positioned themselves to initiate and promote ACP through ongoing and trusted relationships with their patients [23].

Recently, a nurse-led ACP has emerged and demonstrated improvement in the quality of patient-physician end-of-life care communication in patients with COPD in the Netherlands [5], and produced higher plan completion rates in Australia [3]. An American study of a nurse-led patient-centered ACP in primary care has also demonstrated a promising pilot result with 95% (*n* = 38) of participating patients completing ACP documentation [24]. An Australian study of the experiences of 13 patients who participated in the nurse-led ACP program in a general practice found that discussions about working through ideas around end-of-life care with nurses were helpful, with participants perceiving the nurses as being knowledgeable and providing objective professional advice [4]. A nurse-led and facilitated ACP intervention also demonstrated a higher ACP uptake by patients with respiratory malignancy, COPD or interstitial lung disease in a metropolitan hospital in Australia [7].

Previous studies have provided promising results and insights about evolving roles for nurses in ACP amongst other health care professionals. However, it must be noted that the population is narrowly targeted to patients with malignancy, COPD or interstitial lung disease [3, 7] in urban/metropolitan hospitals [5, 7, 21], in either General Practice [3, 4], primary care [24], or residential aged care [22]. The sample sizes of those nurse-led and/or facilitated intervention programs vary from 13 [4], 38 [24], 54 [7] to 3017 [3]. The findings from theses studies indicate that nurses’ roles and responsiblities in implemenation of ACP intervention are still in their infancy and warrant further studies.

## Methods

We conducted a multi-site quasi-experimental study involving 16 sites (8 intervention and 8 control) in two local health districts (LHDs) to investigate the effects of a nurse-led normalised ACP (NACP) service as a routine practice in acute and community settings with the use of specially trained Registered Nurses (RNs). The study protocol for this quasi-experimental trial is published elsewhere [25]. The aim of the qualitative arm of the study was to investigate the experiences of nurses with the implementation of the nurse-led NACP service.

### Theoretical framework

The Normalisation Process Theory (NPT) was used as a theoretical framework in the implementation of a nurse-led NACP service. NPT focuses on the social organisation of work (implementation); making practices routine elements of everyday life (embedding) and sustaining embedded practices in their social context (integration). NPT focuses on what people do in the process of implementation. Four core constructs of NPT are often discussed for actors (personnel) who work around the practices, which include; coherence, cognitive participation, collective action and reflexive monitoring [26–29]. The details of how NPT was applied is published elsewhere [25].

**Design:** a qualitative descriptive approach using a semi-structured interview and weekly debriefing meeting.

**Settings:** The nurse-led NACP was implemented at two hospital settings and two community health care settings in New South Wales (NSW), Australia for a 6 months duration.

### Participant selection

**Fig. 1:**
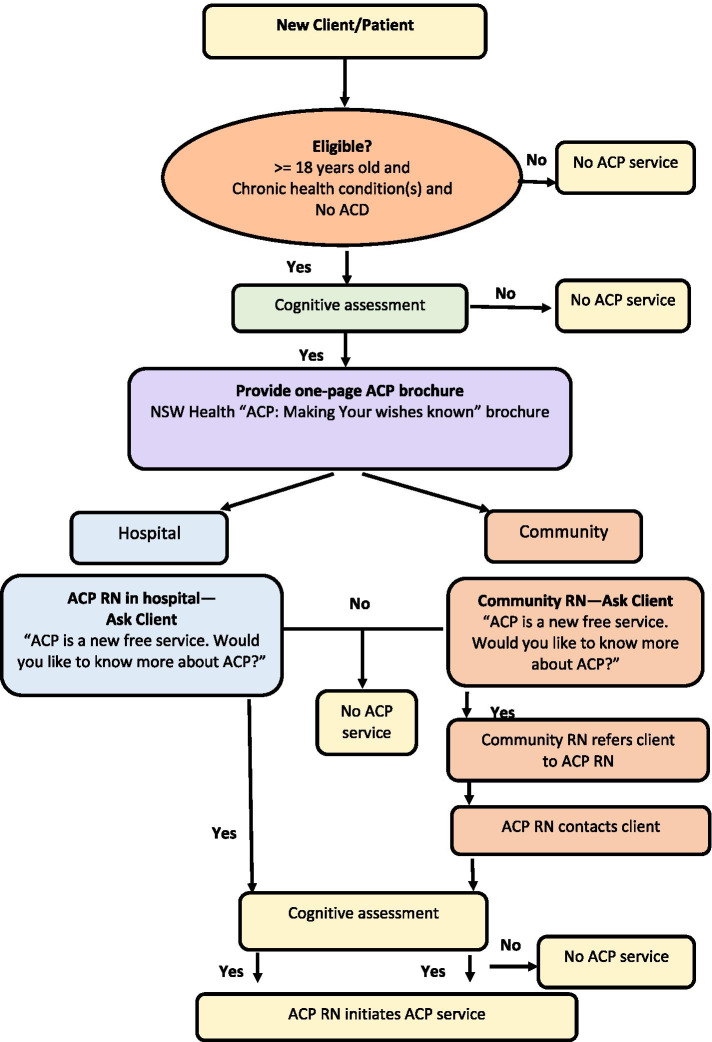
Flowchart: NACP service process

Four purposefully recruited and trained RNs who did not have any relationship with the research team previously undertook the role of ACP RNs described in Table 1. NACP service process is presented in Fig. 1.

**Table 1:** Role of ACP RNs

• Assess newly admitted people with chronic diseases and their nominated substitute-decision makers (SDMs) who meet the initial screening criteria for English proficiency, cognitive impairment and acute episode of mental illness. • Assess those who meet the initial screening criteria for further cognitive assessment using Montreal Cognitive Assessment (MOCA) and Mini-Mental State Examination (MMSE), if necessary, prior to initiation of the conversation. • Explore the person’s knowledge, attitude and desire to participate in ACP. • Identify whom should be involved in ACP conversations. • Initiate and facilitate a series of conversations with clients, family and clinical team to assist clients in completing their ^a^Advance Care Directive (ACD) [1] as they wish by clarifying the person’s understanding of diagnosis, prognosis and preferences for treatment options and place of care. • Liaise with treating healthcare professionals, existing palliative care service, health care interpreters, Aboriginal Health Worker or appropriate Cultural Support Person as required. • The conversations will be captured in an ACD form and/or an ACP Conversation Card (which is a size of business card when folded and which will be carried in participating client’s wallet/purse to alert ambulance officers and other treating health care professionals of the existence and location of completed ACDs. See Additional file 1. NACP Conversation Card). • Upload/store/file/locate a completed Conversation Card in clients’ wallet/purse. • Upload/store/file/locate a completed ACD to ‘^b^My Health Record’, medical records, and elsewhere as appropriate, to be accessible to guide ongoing care. • Participate in weekly peer support/mentoring/troubleshooting with project Leads via online or face to face meeting.	

^a^An Advance Care Directive is a legally binding document made by a legally capable person about the person’s specific wishes and preferences for future care. ^b^My Health Record is an online summary of one’s key health information which facilitates Australia’s need to a connected healthcare system

The four ACP RNs completed a five-day training program online and face-to-face. The Template for Intervention Description and Replication (TIDieR) checklist [30] is used, although it is not an intervention but a training program, to improve the completeness of reporting and the replicability of training program (See Table 2. A summary of a 5-day training program). All four ACP RNs were invited to participate in a semi-structured interview and weekly debriefing meetings.

**Table 2:** A summary of a 5-day training program modified from TIDieR [30]

What: Name	Why: Rationale	What: Materials & Procedures	Who provided	How and where	When and how much	Tailoring & modifications
**Needs assessment**	To identify ACP RNs’ current understanding of ACP and to assess their learning needs	A need assessment survey developed by research team	ACP experts in research team	Paper-based in an orientation session	Pre and post, 5 demographic questions and 15 questions about ACP topics	N/A
**Roles and responsibilities**	To establish shared understanding of roles and responsibilities	Print outs, oral presentation by team & discussion with all	ACP experts in research team	Oral presentation and discussion in a training room	Day 1, Session 2–1 h	Roles and responsibilities were explained and discussed, and all questions were answered.
**ACP & ACD**	To gain knowledge about ACP & ACD including capacity, legal and ethical aspects	NSW ACP materials and ACD booklet, oral presentation & discussion	ACP experts in research team	Oral presentation and discussion in a training room	Day 1, Session 3–1.5 h	All questions were answered.
		‘Supporting health professionals in ACP’ – Four modules online: Mandatory	Health Education and Training (HETI)	Completion of four online modules by week 1	2 h for four modules online, complete once	ACP RNs were self-directed to complete four modules online at their own pace.
		Three modules online developed by ACP Australia: Mandatory	ACP Australia	Completion of four online modules by week 1	1.5 h for three modules online, complete once	ACP RNs were self-directed to complete three modules online at their own pace.
		Three modules online developed by ACP Australia: Recommended	ACP Australia	Completion of four online modules by week 2	1.5 h for three modules online, complete once	ACP RNs were self-directed to complete three modules at their own pace.
	To apply the knowledge gained about ACP & ACD	Role play with four scenarios	Four ACP RNs and ACP experts in research team	Role play and discussion in a training room and in participating wards/community centres	Day 3, Session 2–1.5 h	Role play with four scenarios generated meaningful questions and discussions which were tailored to their level of understanding.
**Grief, loss & relief, Gerotranscendence**	To understand and underpin ACP with other relevant concepts	Published literature	ACP experts in research team	Reading and discussion in a training room	Day 2, Session 3 and as necessary throughout the intervention period.	ACP RNs were self-directed to read literature at their own pace. Meaningful questions and discussions were tailored to their understanding.
**Forms and logs**	To fulfil documentation requirements for ACP and research project	Capacity assessment, referral logs, consent logs, conversation logs, ACD logs, oral presentation & discussion	ACP experts in research team	Oral presentation and discussion in a training room	Day 2, Session 1 and as necessary throughout the intervention period.	ACP RNs were supported by ACP experts in research team how to complete documentation requirements throughout intervention period.
**NACP Service**	To deliver NACP service	NSW ACP materials and ACD booklet	Four ACP RNs	In participating wards/community centres	Day 4 & 5: a half-day NACP service delivery and a half-day debriefing and troubleshooting	Four ACP RNs were encouraged to deliver NACP service at their own confidence and pace. Meaningful questions and discussions were tailored to their cases and understanding.
**Ongoing feedback and support**	To ensure the fidelity of the intervention and support four ACP RNs	NSW ACP materials and ACD booklet, referral logs, consent logs, conversation logs, ACD logs, oral discussion	Four ACP RNs and ACP experts in research team	Oral discussion in a training room, & phone meeting	Weekly debriefing and troubleshooting during the intervention period	Meaningful questions and discussions were tailored to their cases and understanding.

### Data collection and analysis

Face-To-Face semi-structured interviews with four RNs were conducted by SJ at the conclusion of the intervention. The minutes of weekly debriefing meetings with the research team (SJ, SO) were also used. Interviews took about 30–60 min. The interview guide is presented in Additional file 2. The interviews were audio-recorded and transcribed verbatim by an independent transcribing service and entered into a qualitative data analysis software. Codes and categories were developed by listening to interviews and reading transcripts by two of the researchers (SO, PC). Any disagreements were solved by discussion with another researcher (SJ). The data collected through interviews and debriefing meetings provided sufficient data to achieve saturation and enabled a full description of the experiences of ACP RNs. All collected data were analysed using thematic content analysis because the data obtained from the transcripts are narratives, stories and experiences of nurses [31]. This method involved reading, browsing, reflection, coding, validation of codes and searching for emerging patterns in the data. This process also involved making comparisons and asking questions in relation to research questions. All participants reviewed their transcripts and none made any edits. Disagreements in these processes were resolved by consensus. Quotes from participants were used to illustrate the themes and to keep the interpretation closely linked to the original data. The Consolidated Criteria for Reporting Qualitative Studies (COREQ [32]; has been used in reporting this study (Additional file 3. COREQ checklist).

### Ethics statement

The study was approved by the Hunter New England Human Research Ethics Committee (Approval no. 17/12/13/4.16). Informed written consent was sought and obtained for uptake of NACP service, and voluntary participation was ensured. The study was conducted in accordance with the National Health and Medical Research Council’s National Statement on Ethical Conduct in Human Research (2007), and under the governance of the Human Research Ethics Committee (HREC) at the University of Newcastle and the two Local Health Districts.

### Findings

All four ACP RNs were females with a mean age of 43 years old. Their nursing experience ranged 2 to 25 years but they had minimal experiences with ACP and had not attended any education about ACP previously. The experiences of ACP RNs with the implementation of NACP service as a routine service was described in four inter-related themes; 1) Embracing NACP service; 2) Enablers and barriers related to patients and health professionals; 3) Enablers and barriers related to ACP RNs; and 4) What it means to be an ACP RN.

### Embracing NACP service

The ACP RNs themselves had limited awareness and little experience in ACP before role commencement.

"I really didn't know a great deal about it (ACP) and yeah because of that I wouldn't talk about it too much (to patients before). If there were times where a patient clearly needed to have something put in place, you'd sort of sitting there and stew and sort of think well how do I approach this subject with the patient, how do I talk to the family about it." ACP RN A

Despite lack of knowledge, ACP RNs recognised ACP as being part of person-centred care that respects patient autonomy.

"ACP is a necessary and very important aspect of patient care and ensures we are caring for our patients holistically. There is a lot of stigma attached to Advanced Care Planning for patients, families and professionals. When Advanced Care Planning is done and in my experience, it makes the death of a person as good a death as possible for all involved. I am very excited to be a part of this project as I hope Advanced Care Planning will become a normal part of health care." ACP RN A

"I feel strongly about people having the right information and control over their care when they are unable to speak for themselves”. ACP RN D

The ACP RNs also reported that ACP was not a routine practice in the study setting. It was necessary for them to introduce the ACP service to the existing health professionals and patients in the care settings. They identified that the normalisation of the ACP service required their effort to integrate the ACP as a standard care provision.

"I see the need to have Advance Care Planning in place. I saw cases where there was nothing…. Social workers, just from what I observed with the notes, they'll give out information about Enduring Guardian, but it doesn't go any further. And then the doctors only talk about the end of life stuff when it is at the end of life and what do you want. And so I do definitely see there being a role there..." ACP RN D

Over the intervention period, the ACP RNs have fully embraced and committed to NACP service because of their better understanding of the process of ACP.

"It (ACP) needs to be done but just having that confidence and having the knowledge to say I know what I'm talking about.… Now after the project, I don't have a problem talking to people about it (be)cause again you have that knowledge, you know what you're talking about. It's just a matter of sussing out the patient and how to deliver that information gels with them." ACP RN A

### Enablers and barriers to NACP service related to patients and health professionals

During the intervention the ACP RNs encountered enablers and barriers related to patients and health professionals. The level of acceptance of ACP and willingness to complete ACD were impacted by patient’s understanding of their illness, past death and dying related experiences and other nurses’ personal values or beliefs towards ACP.

Some patients were not interested in, or capable of, participating in the NACP Service process.

"(Some patients were) not interested. Sort of like an ostrich, bury your head in the sand and not face those issues (be)cause it's too hard, that sort of mentality. Some people just found it absolutely too confronting and they just don't want to talk about that sort of thing at all." ACP RN A

On the other hand, others welcomed and were enthusiastic about the ACP. For example, one couple even took the idea of ACP to their friends in a BBQ, this led to all of their friends wanting to take up ACP as well. ACP RN B reported that

"There are people out there screaming at me, "Come and speak at my …Club". Even yesterday, (a patient) and his wife is saying, "All their partners are doing this too", as you know, they're doing a (Advance Care) directive." ACP RN B

Past death and dying experiences of a member of the family, or near dying experiences among the patients, could facilitate or prevent participation in ACP conversations.

"Like, one of women, she'd had some near-death, close death experiences with it (asthma) and she was keen to have something in place." ACP RN D

"She's gone through terrible troubles with her mum, had massive family arguments and she said it was a horrible time for everyone so that was the main reason why she wanted to do one." ACP RN A

So a lot of people from, elderly people from M Community Care Services feel that way. They just can’t cope with another thing. Other ones will be, a lot of the others … they’ll seem like yeah it’s a good idea but then they, it’s not as important as everything else that they’ve got going on. So they’re the kind of people that it’s not as important. I have had visits with people who have had the opposite. So one lady who her mother was told at the age of 70 well that’s the end for her, well she had some sort of treatment I can’t recall what it was but something, she lived for another 20 years. So that woman who was in her 90 s refuses advanced care directives because she believes that she would be signing her own death warrant. That happens a fair bit. ACP RN B

Concern about the impact of major illness on family members was associated with willingness to uptake of ACP. All ACP RNs reported similarly that

"They were prepared to take that step and make it easier for other family members …they saw this is a good idea and they would do it…." ACP RN D

For the nurses that ACP RNs worked with, their beliefs, knowledge, behaviour and attitudes about ACP impacted the implementation of ACP in routine practice. Some were more aware of and accepting of ACP.

"*There was a little bit of resistance. One (community) RN stated her views on it and felt that ACDs were so final. She did indicate that she doesn't really agree with them (ACD and ACP) and she doesn't hand them (the ACP flyer) out to everyone and get them to do it." ACP RN C*

While all ACP RNs described some degree of resistance in the implementation of the ACP service among the existing staff, others were committed to implementation of the ACP service as a routine practice. ACP RN B further appreciated a community nurse’s commitment to ACP.

"I had one male nurse in particular who is absolutely brilliant and he gets the prize for the most referrals given to me at the end. Most nurses don't think about it. You constantly have to ask next time you see Mrs such and such can you please ask (if they want the ACP Nurse to visit)." ACP RN B

### Enablers and barriers related to ACP RNs

The ACP RNs shared what worked well and what challenged them in delivering NACP service, and these included; 1) the training program, and weekly debriefing and troubleshooting meetings, 2) the referral process for the NACP service in community settings, 3) everyone loved the Conversation Card, 4) let it take its course in a series of conversations during the ACP process, and 5) is hospital right place to do ACP?

Firstly, all ACP RNs stated that ***the training program enabled them to gain the knowledge and skills to deliver the NACP service***. The ACP RNs also mentioned gaining their confidence as they implemented the ACP service.

"I have not discussed ACP/ACD in depth due to lack of knowledge and time constraints before. I have now completed 3 days of ACP/ACD training and am starting to feel more confident as my knowledge increases. "ACP RN B

"After completing the ACP training and getting to talk to some patients about it, I have become more confident in discussing this topic with patients and their families." ACP RN A

Central to supporting the ACP RNs in sustaining the ACP practice was ***a weekly debriefing and troubleshooting meeting with the ACP experts*** (SJ, SO) in the research team. This enabled the exploration of issues around service delivery as they arose and consistency of advice about challenging cases.

*“The weekly meetings were fantastic that was absolutely necessary. …Absolutely. (We) are doing the same job but it’s chalk and cheese as well so it was really good to hear how she was going… particularly because as I say it’s not been done before so we were really feeling our way to make it work, ... it was good to be able to touch base once a week to say this is where I’m at challenging cases, absolutely.” ACP RN B.*

Secondly, the ACP RNs mentioned that ***the referral process to the ACP service in community setting*** was a challenge as it was dependant on the existing staff’s belief and attitudes about ACP.

"The first nurse was one of the ones who would not refer and when the second nurse happened to mention to me 'oh this gentleman has said that he'd be really good and he's said yes', she (the first nurse) actually turned around and said 'you can't harass that man, he's got enough going on, he's got cancer…." ACP RN B

Thirdly, the ACP RNs commented ***how useful the ACP Conversation Card was*** to initiate the conversations and to capture iterative patient conversations.

"The patients have absolutely loved it (Conversation Card). As soon as it's all complete and everything, they fold it up and straight in their purse it goes." ACP RN A

ACP RN A further explained that other health professionals such as social workers accepted the ACP conversation Card well, saying *“They love it, absolutely love it. All think it’s awesome and I’m surprised we haven’t got something like sooner, so thumbs up for that one, everybody absolutely loves it.”*

The number and duration of conversations needed for the ACP process surprised the ACP RNs. The ACP RNs realised ***it was important to let it take its course and to let them (patients) reflect their life and transcend***. The sequence or topic of conversations with the clients/patients for ACP were similar among all ACP RNs. In general, the first conversation was to build rapport with the patients and introduce ACP to the patients with explanation about the elements of ACP such as ACD, substitute decision-makers, Enduring Guardians, Power of Attorney, and will. In the second conversation, they explored values and their preference of care using the Conversation Card. Although there were some patients who required more than three conversations, it was usually at the third conversation where they would finalise the ACD with all the signatures which would make the ACD legally binding. The conversations were spaced around 1 to 2 weeks apart to allow them to talk with family members, reflect on their situations, and experience gerotranscendence during a series of conversations.

"they have gone through life and sort of … it’s just part of all is that you’re going to die one day and preparing for that… a lot of them had the experience of reflecting their life, how they lived and transcended, a lot of them. They would even say ‘I’ve lived my life’, you know that was a common quote… A lot of people had children died or their partner had died and they'd reflect on that. Had a few tears from a few clients and things at the first visit. People needed time to reflect on it. Then I would introduce the conversation card because people would start to say the (ACD) document. And then I'd go through every section, explain what it meant, ask are there any questions, and leave it with them. I'd say to them to take time as long as you need. I would make the follow-up appointment then or I would say, well how about I'll give you a call in a week or two, depending on what they'd indicated. And follow-up as appropriate." ACP RN C

The last aspect of the ACP RNs’ experiences was related to considering ***‘if hospital was the right place to do ACP?’*** The experiences of providing an ACP service were markedly different for each ACP RN between hospital and community settings. The two ACP RNs who were delivering ACP service in hospitals found that the hospitals were not a place for effectively and meaningfully providing ACP service. The two ACP RNs in hospital settings expressed their disappointment with low uptake of NACP service and described the challenges in relation to place, space, time, and context. An example of these challenges was demonstrated in the following interview excerpt,

"It was just very difficult seeing patients. They were either sleeping (or)… It would be chockers with staff… it was noisy, the lack of privacy. We couldn't hear each other. It was that hard… just a lot of talking because everybody else had visitors and doctors … so you can't have a discussion about something like this (ACP) when there's people screaming around you. You go around, okay, I can't see them this time. Go round again, still can't see them." ACP RN D

In contrast, both ACP RN B and ACP RN C who worked in community settings had better experiences in delivering the ACP service.

"If you can offer that (ACP) service to people in the community while they're well, they appreciate it. They really appreciate because I've taken the time, I'm sitting there getting to know who they are, (and) we've met their family. It's relaxed, there's not a hurry. It is a tough topic to think about and discuss so you make it as light and as comfortable as possible and that happens in the community. … It needs to be in communities." ACP RN B

### What it means to be an ACP RN

The ACP RNs shared what it means to be an ACP RN. They strongly voiced their satisfaction with their role with positive expressions such as ‘*confident*’, ‘*sense of achievement*’, ‘*making differences*’, ‘*happy to see them (patients) in control*’, ‘*give power back to them*’ and ‘*opportunity to their say*’.

All ACP RNs felt that they contributed to achieving quality patient care delivery, especially end of life care.

"They were the most, for me personally, as the job, they were the most enjoyable…. I've done something. this is something that you can do (for them), take some power back to themselves regarding their health… It can be difficult, it's stressful in those points in time, but you can help (them) to have this (ACP). I've encouraged them to talk to their relatives, their children, partners, to open up the conversation." ACP RN D

All the ACP RNs recommended a continuing designated ACP RN because there is a lack of knowledge and skills among health professionals who do not consider this as part of their role.

"There was nothing and it was just the staff were having a really difficult time trying to sort this (ACP) out. …. If it was a (dedicated) role full-time, I think the doctors and social workers could refer to that (my) position because I can see where there is nobody directly dealing with this (ACP)." ACP RN D

## Discussion

The experiences of ACP RNs included embracing NACP service, enablers and barriers of uptake of ACP service, strategies that assisted them in delivering ACP service, and what it meant for them to be an ACP RN.

### ACP RNs do not have to be a specialist to deliver the NACP service, but …

Consistent with other studies [4, 5, 11], the findings from the experiences of the ACP RNs demonstrate that they were able to deliver the NACP service and increased the uptake of ACP, by raising awareness of ACP among the patients and family members, and supporting them through the process. In previous studies, nurses who facilitated ACP were senior nurses [7], respiratory nurse specialist [5], and nurses with a mean of 6 years experience in the general practice setting [4]. The ACP RNs in this study were not a senior or specialised personnel (e.g. Clinical Nurse Consultant (CNC), Clinical Nurse Specialist), but were general RNs who should be available at various settings. This study adds new evidence that ACP facilitators do not have to be a clinical specialist. However, the ACP facilitators in previous studies all received additional training which varied from a 2-day training [5] to a full-day workshop [7]. It is not specified what trainings were provided in other studies [3, 4]. Although Miller et al. [4] provided some insight about the training workshop, there is no detailed report on what the training entailed. This study provided the detailed a 5-day training program in Table 2. This study demonstrated that purposely designed face-to-face small group training, supported by on-line resources, was sufficient to equip RNs to deliver an ACP service. Use of validated resources, such as the NSW Health ACP Flyer and Workbook or standard recording formats, such as the Conversation Card, facilitated consistent delivery of ACP. It is also important to note that ACP facilitators were also supported regularly and as necessary by a CNC [4] and ACP experts in this study to improve their competency and confidence [9]. The findings from previous studies and this study indicate that a nurse-led ACP service has been emerging and has contributed to increased uptake of ACP. Although it is not explicitly reported, an additional training and ongoing support for general RNs are necessary. The content, duration and mode of delivery of training program for ACP RNs warrant further implementation studies in various contexts.

### The normalisation of ACP is definately a challenge for some

ACP is a relatively new concept in the experiences of the ACP RNs. It is often a challenge for patients and community members, indeed all health professionals, including the ACP RNs themselves. There is a gap between a policy perspective of the value of and need for ACP, and the contextual reality of health care settings. Yet ACP is promoted, even enforced by health care policies and health organisations [8, 19]. While a systematic review covering 1660 original articles highlights that legislation and institutional policies influence ACP development [9], the ACP practiced is ad hoc, fragmented and inconsistent. This reivew provides evidence that current practice does not place a high value on ACP activity except where it is in response to a critical incident or serious clinical event. A degree of cognitive dissonance can explain a clincian’s perception and behavour. While many staff, when asked, expressed support for more ACP, many of them were unable or unwilling to change behaviour to incorporate the provision of ACP information as a routine activity into their role. As effective ACP requires a time investment through conversations that may need to be iterative, the expectation that hospital clinicians who see themselves as very busy and under stress will be able to step into more ACP related tasks is unlikely to be realised broadly. While merit can be seen in arguments for both an “everybody’s business” and a “specialised few” approach to ACP service provision the “right” normal may justifiably be different in different care settings. Further, not all consumers (patients) chose to participate in the ACP service, but those who did participate reported value in the ACP process.

The importance of setting to deliver the ACP service is discussed widely in the literature such as outpatient setting [33], nursing homes [22, 34], and inpatient hospital settings [35]. Similar to the findings of Batchelor et al. [20], the ACP RNs of this study echo the ACP service at the community setting is more effective than in the inpatient hospital setting. However, the complexity of ACP processes, and in particular the variations in attitudes of both health professionals and patients towards these processes, indicate the need for ACP Services that are robust and adaptive.

### Limitations

The interviews of ACP RNS were conducted by one (SJ) of the ACP experts, and the weekly debriefing meetings were conducted two (SJ & SO) of the ACP experts who provided a large part of the training. Given that the interviewers were the people who developed and delivered the training and oversaw the implementation there were concerns that the ACP RNs might not feel comfortable sharing their experiences. To ensure open and honest feedback from the ACP RNs, during the debriefing meetings, the ACP RNs were encouraged to share their experiences to resolve any challenges that they encountered collaboratively as part of trouble-shooting exercise. The interviews were conducted 1 to 2 weeks after the completion of the intervention to ensure there was no influence, coercion and pressure during intervention period. The four ACP RNs shared their experiences as a reflection activity during interviews. In fact, the ACP RNs did provide critical or negative opinions, particularly about the training (e.g. discomfort with role play). The questions asked (Additional file 2) were open and inviting. The direct quotes about enablers and barriers illustrated in the findings represent the open and honest views of the ACP RNs and demonstrate that the basis of the findings is drawn from the voices of the ACP RNs. We neither have objective evaluations of the RNs’ performance/skills/competencies nor have information about the usual staff. However, the patients’ opinion about the ACP RNs as leaders of ACP is collected and will be published elsewhere. Another limitation is that the study did not focus on long term impacts on professional practice nor did it consider the way ACP impacted upon future care outcomes for users of the service as the study focussed on RN experience in the establishment of an RN led ACP service.

## Conclusion

Believing in and commitment to the ACP service by all actors (the ACP RNs, patients and their families, the existing health care professionals) are essential for the implementation of the ACP service. The successful implementation of the ACP service can be determined by the challenges and enablers such as contexts, the process and tools that are used to implement the ACP service. The ACP RNs highlight the need to address those challenges and enablers to succeed in the implementation of a nurse-led ACP service.

## Supplementary Information

**Additional file 1.**

**Additional file 2.** Interview guide.

**Additional file 3.** Consolidated criteria for reporting qualitative studies (COREQ): 32-item checklist.

## Data Availability

The dataset supporting the conclusions of this article is included within the article and its additional files.

## Abbreviations

ACP: Advance Care Planning
NACP: Normalised Advance Care Planning
NPT: Normalisation Process Theory
NSW: New South Wales; RN: Registered Nurses
