# Supplementary material for: Nurse-led normalised advance care planning service in hospital and community health settings: a qualitative study
Source: BMC Palliat Care. 2021 Sep 9;20:139. doi: 10.1186/s12904-021-00835-x (PMC8431845; doi:10.1186/s12904-021-00835-x)
Supplement: Supplementary file 1 — Additional file 1. [file 12904_2021_835_MOESM1_ESM.pdf]

CONVERSATION NO. ____

DATE: ____

PEOPLE PRESENT DURING CONVERSATION

Name 1: ____

Role: ____

Signature: ____

Name 2: ____

Role: ____

Signature: ____

Name 3: ____

Role: ____

Signature: ____

CONVERSATION NO. ____

DATE: ____

PEOPLE PRESENT DURING CONVERSATION

Name 1: ____

Role: ____

Signature: ____

Name 2: ____

Role: ____

Signature: ____

Name 3: ____

Role: ____

Signature: ____

ADVANCE CARE PLANS FOR MY CARE

I have discussed plans for my care and/or who should make medical decisions for me if I can't speak for myself.

Please read the information inside.

CONVERSATIONS MAY INCLUDE, BUT ARE NOT LIMITED TO:

My values

My directions for care

Specific requests

DATES THIS FORM WAS REVIEWED TO CHECK ITS CURRENCY:

My name: ____

Date reviewed: ____

Signature: ____

Date reviewed: ____

Signature: ____

Date reviewed: ____

Signature: ____

Health Central Coast Local Health District

Health Hunter New England Local Health District

NSW GOVERNMENT

THE UNIVERSITY OF NEWCASTLE AUSTRALIA

Name: ____

DoB: ____

Address: ____

Phone Number: ____

Name of GP: ____

GP Tel: ____

GP Informed Yes ☐ No ☐

INSTRUCTIONS FOR HEALTH CARE PROFESSIONALS:

This represents part of an ethically approved research study and is a working document, on Advance Care Planning, to be used as a guide.

Please consider all content, the most recent entry takes precedent.

WHO SHOULD MY DOCTOR TALK TO?

My medical decision maker(s)

Name 1:

Relationship:

Tel:

Name 2:

Relationship:

Tel:

Formally legally appointed

Yes ☐ No ☐

ESSENTIAL SPIRITUAL/ CULTURAL/ RELIGIOUS CONTACT PERSON

I would also like the following person to be involved in discussions about my health care:

Name 1:

Relationship:

Tel:

Name 2:

Relationship:

Tel:

OTHER IMPORTANT CONTACT(S)

I would also like the following person to be involved in discussions about my health care:

Name 1:

Role:

Tel:

Name 2:

Role:

Tel:

CONVERSATION NO.

DATE:

PEOPLE PRESENT DURING CONVERSATION

Name 1:

Role:

Signature:

Name 2:

Role:

Signature:

Name 3:

Role:

Signature:

CONVERSATION NO.

DATE:

PEOPLE PRESENT DURING CONVERSATION

Name 1:

Role:

Signature:

Name 2:

Role:

Signature:

Name 3:

Role:

Signature:
