# Supplementary material for: Nurse-led normalised advance care planning service in hospital and community health settings: a qualitative study
Source: BMC Palliat Care. 2021 Sep 9;20:139. doi: 10.1186/s12904-021-00835-x (PMC8431845; doi:10.1186/s12904-021-00835-x)
Supplement: Supplementary file 2 — Additional file 2. Interview guide. [file 12904_2021_835_MOESM2_ESM.docx]

**Additional file 2. Interview guide**

1. What did you feel and learn during training? Was it sufficient? was there any other support you needed?
2. In your role, what worked well? and why?
3. In your role, what did not work so well? and why?
4. What other health care professionals should be involved in ACP service?
5. In your opinion, who were those people who clearly & definitely declined your service and why did they decline?
6. In your opinion, who were those people who clearly & definitely welcome and accepted your service and why did they accept?
7. How would you describe your experiences of NACP in initiation, conversations, documentation, storage, and execution?
8. How do you think people find about conversation card? Any comments and suggestions?
9. How do you think the ‘normalisation’ played or worked?
10. How do you think the ‘gerotanscendence’ played or worked?
11. What were the challenges you have had?
12. What were the enablers you have had? What and who helped you?
13. What were the strategies that you have used?
14. What difference do you think you have made with your role?
15. What do you think it needs to happen to ACP service and RN ACP Facilitator?
16. Any other comments or suggestions related to your experiences with Advance Care Planning Facilitator and ACP.
