# Supplementary material for: Nurse-led normalised advance care planning service in hospital and community health settings: a qualitative study
Source: BMC Palliat Care. 2021 Sep 9;20:139. doi: 10.1186/s12904-021-00835-x (PMC8431845; doi:10.1186/s12904-021-00835-x)
Supplement: Supplementary file 3 — Additional file 3. Consolidated criteria for reporting qualitative studies (COREQ): 32-item checklist. [file 12904_2021_835_MOESM3_ESM.docx]

**Additional file 3.**

Consolidated criteria for reporting qualitative studies (COREQ): 32-item checklist

| **No** | **Item / Page No** | **Guide questions/description** |
| --- | --- | --- |
| **Domain 1: Research team and reflexivity** |  |  |
| Personal Characteristics |  |  |
| 1. | Interviewer/facilitator/ 7 | Which author/s conducted the interview or focus group? |
| 2. | Credentials / Title page | What were the researcher's credentials? *E.g. PhD, MD* |
| 3. | Occupation / Title page | What was their occupation at the time of the study? |
| 4. | Gender / N/A | Was the researcher male or female? |
| 5. | Experience and training / Title page | What experience or training did the researcher have? |
| Relationship with participants |  |  |
| 6. | Relationship established / 5 | Was a relationship established prior to study commencement? |
| 7. | Participant knowledge of the interviewer / 5,7 | What did the participants know about the researcher? e*.g. personal goals, reasons for doing the research* |
| 8. | Interviewer characteristics / Title page, 7 | What characteristics were reported about the interviewer/facilitator? e.g. *Bias, assumptions, reasons and interests in the research topic* |
| **Domain 2: study design** |  |  |
| Theoretical framework |  |  |
| 9. | Methodological orientation and Theory / 4 | What methodological orientation was stated to underpin the study? *e.g. grounded theory, discourse analysis, ethnography, phenomenology, content analysis* |
| Participant selection |  |  |
| 10. | Sampling /Abstract, 5 | How were participants selected? *e.g. purposive, convenience, consecutive, snowball* |
| 11. | Method of approach / 4, 7 | How were participants approached? e*.g. face-to-face, telephone, mail, email* |
| 12. | Sample size / 4,7 | How many participants were in the study? |
| 13. | Non-participation / NA | How many people refused to participate or dropped out? Reasons? |
| Setting |  |  |
| 14. | Setting of data collection / 4 | Where was the data collected? e*.g. home, clinic, workplace* |
| 15. | Presence of non-participants / N/A | Was anyone else present besides the participants and researchers? |
| 16. | Description of sample / 8 | What are the important characteristics of the sample? *e.g. demographic data, date* |
| Data collection |  |  |
| 17. | Interview guide / Additional file 2 | Were questions, prompts, guides provided by the authors? Was it pilot tested? |
| 18. | Repeat interviews / NA | Were repeat interviews carried out? If yes, how many? |
| 19. | Audio/visual recording / 7 | Did the research use audio or visual recording to collect the data? |
| 20. | Field notes / NA | Were field notes made during and/or after the interview or focus group? |
| 21. | Duration / 7 | What was the duration of the interviews or focus group? |
| 22. | Data saturation / 7 | Was data saturation discussed? |
| 23. | Transcripts returned / N/A | Were transcripts returned to participants for comment and/or correction? |
| **Domain 3: analysis and findings** |  |  |
| Data analysis |  |  |
| 24. | Number of data coders / 7 | How many data coders coded the data? |
| 25. | Description of the coding tree / N/A | Did authors provide a description of the coding tree? |
| 26. | Derivation of themes / N/A | Were themes identified in advance or derived from the data? |
| 27. | Software / N/A | What software, if applicable, was used to manage the data? |
| 28. | Participant checking / 7 | Did participants provide feedback on the findings? |
| Reporting |  |  |
| 29. | Quotations presented / 8 - 13 | Were participant quotations presented to illustrate the themes / findings? Was each quotation identified? e*.g. participant number* |
| 30. | Data and findings consistent / 8 - 13 | Was there consistency between the data presented and the findings? |
| 31. | Clarity of major themes / 8 - 13 | Were major themes clearly presented in the findings? |
| 32. | Clarity of minor themes / 8 - 13 | Is there a description of diverse cases or discussion of minor themes? |
